# The comparison of acute phase proteins in experimentally induced canine acute pancreatitis

**DOI:** 10.1186/s12917-026-05409-8

**Published:** 2026-03-10

**Authors:** Eszter Tünde Kanyorszky, Ágnes Sterczer, Zsuzsanna Vizi, Emma Léda Babits, Nándor Balogh, Katalin Lányi, Ferenc Manczur

**Affiliations:** 1 Department of Internal Medicine, University of Veterinary Medicine Budapest, István utca 2, Budapest, 1078 Hungary; 2 Praxislab Kft, Iskola u. 3, Budapest, 1038 Hungary; 3 Department of Food Hygiene, University of Veterinary Medicine Budapest, István utca 2, Budapest, 1078 Hungary

**Keywords:** Acute phase proteins, Albumin, C-reactive protein, Haptoglobin, Hepcidin, Serum amyloid A, Canine pancreatitis, Experimental acute pancreatitis

## Abstract

**Background:**

Acute pancreatitis triggers the production of acute-phase proteins (APPs) in the body, among which C-reactive protein (CRP) is the most extensively studied and widely used for both diagnosis and prognosis. The diagnostic value of other APPs is less well known. Our study aimed to compare six APPs in a canine model of experimentally induced acute pancreatitis (AP). We measured serum CRP, serum amyloid A (SAA), haptoglobin (HAPT), hepcidin-α and -ß (HEPC-α, HEPC-ß), and albumin (ALB) levels in 10 dogs over 8 consecutive days following AP induction with cerulein injections.

**Results:**

During the study, CRP was the most consistent marker for tracking temporal changes in pancreatic enzymes among the studied APPs. We found no correlation between pancreatic enzymes and CRP or SAA levels; however, a significant positive correlation was observed between CRP and SAA. A moderate correlation was identified between serum cholesterol levels and both CRP and SAA concentrations. Albumin exhibited a moderate negative correlation with CRP and SAA. Neither HAPT nor hepcidins correlated with CRP, SAA, or pancreatic enzymes. Serum albumin remained within the reference interval.

**Conclusions:**

Based on our experimental results, CRP remains the most consistent APP for monitoring canine pancreatitis. Our findings did not support the practical usefulness of hepcidins or haptoglobin in tracking pancreatitis in dogs.

## Background

 The acute phase response (APR) is a reaction in humans and animals to injuries such as infection, trauma, necrosis, or neoplasia. The APR occurs as a cascade: injury triggers local acute inflammation. As a secondary systemic response, fever, leukocytosis, complement activation, activation of the kinin-forming pathway, and changes in plasma protein synthesis—including acute-phase proteins (APPs)—may occur [1]. These proinflammatory cytokines stimulate APP production mainly in hepatocytes as part of the APR, although weak expression has also been observed in canine kidney and lung tissues [2–4]. Acute-phase proteins are classified as positive or negative based on changes in their concentrations. Positive APPs increase when inflammatory cytokines are produced in the body. They are further divided into three groups depending on their level of increase. Minor (type I) APPs (e.g., fibrinogen) increase slowly, usually less than twofold. In contrast, moderate (type II) APPs (e.g., haptoglobin) show a 1- to 10-fold rise, with peak levels typically occurring three or more days after the stimulus. Major (type III) APPs (e.g., C-reactive protein—CRP, serum amyloid A—SAA) have negligible basal levels in healthy animals but elevate rapidly to 10- to 1000-fold, often within 24–48 h of the stimulus [2, 5, 6]. Similarly, APPs can be classified based on which cytokines activate their response: type I (e.g., CRP, SAA) is induced by interleukin (IL)-1-like cytokines, while type II (e.g., fibrinogen, HAPT, α1-antitrypsin) is triggered by IL–6–like cytokines. Human studies identify hepcidin as a type II acute-phase protein, showing a 25-fold increase within 8 h, induced by IL-6-like cytokines, similar to fibrinogen and haptoglobin [7]. The exact roles of positive acute-phase proteins are still being researched. Some of these proteins, such as ceruloplasmin and hepcidin, transport various molecules to prevent their loss [8, 9]. Changes in APP levels during pathological conditions may help clinicians monitor disease progression. They may serve as prognostic tools, as the magnitude and duration of the APR reflect the severity of inflammation [5].

As mentioned, CRP in dogs is part of the major acute phase protein (APP) group and has high diagnostic potential. Several studies have explored and confirmed this potential, leading to its prominent role in everyday clinical use across various fields of canine medicine, including discospondylitis, heartworm disease, idiopathic polyarthritis, and mammary tumors [10–13]. CRP, along with SAA and HAPT, also shows a significant increase in cases of acute bacterial pneumonia and can be used to monitor the effectiveness of antimicrobial treatment and to determine the optimal time to discontinue antibiotics [14]. Another important APP, SAA, has been increasingly used in dogs recently. It has been measured in systemic inflammation across various diseases: e.g., pyometra, tumors, babesiosis, bordetellosis, pyometra, and apical periodontitis, as well as conditions such as changes during estrus or post-operatively after tibial plateau leveling osteotomy, and it correlates well with CRP [15–24]. Along with CRP, SAA is also a useful tool for diagnosing acute bacterial pneumonia. Because of its broader range and more significant quantitative changes, it is generally better at monitoring disease progression than CRP and HAPT [14]. Lowrie et al. (2009) found similar results when studying APPs (including CRP, SAA, and HAPT) in dogs with steroid-responsive meningitis-arteritis [25]. CRP and SAA showed significant increases, with the latter’s serum levels rising several-fold, thereby enhancing diagnostic potential. In addition to diagnosing disease, these two parameters may also help monitor its progression, as their levels tend to decrease after treatment begins [25]. Christensen et al. (2012) tested an automated latex agglutination turbidimetric (LAT) immunoassay based on a human monoclonal SAA antibody in several species (horse, dog, cat) for its analytical utility [26]. They found it reliable across all three species [26]. Additionally, LAT enables rapid determination of SAA and demonstrates that SAA is a sensitive and specific marker of systemic inflammation in dogs, with less influence from the animal’s stress level [27]. Christensen et al. (2014) published additional results from a retrospective analysis of blood serum from 500 dogs. They confirmed that SAA, due to its wider variation and inherently higher concentration, already detects minor pathological changes and thus has greater diagnostic potential than CRP [15]. Two veterinary studies found that normal SAA serum concentrations were mostly below the lower detection limit in healthy dogs [28].

Haptoglobin (HAPT) is an alpha2-globulin mainly responsible for binding free hemoglobin (Hgb). Its affinity for Hb enables measurement of its concentration using spectrophotometric methods. A prospective case-control study using ELISA reported a median haptoglobin concentration of 0.86 g/L (IQR: 1.1 g/L) in healthy dogs, which aligns with the laboratory normal range of 0.3–3.5 mg/L [29, 30]. Haptoglobin is classified as a moderate acute-phase protein (APP) and is not expected to increase significantly during the acute-phase response (APR). The study by Dabrowski et al. (2009) confirms that CRP and SAA in dogs respond more quickly than HAPT in bitches with pyometra [31]. Nonetheless, several studies have investigated serum haptoglobin levels in various disease conditions [32–36].

Hepcidins are peptides with both antifungal and antibacterial activities, and they also serve as key hormones that regulate iron metabolism [37, 38]. Canine hepcidin was first identified in 2004 [4]. However, in 2019, Mead et al. discovered a similar 25-amino-acid hepcidin molecule, which differed in two amino acids compared to the original canine hepcidin-25 [39]. Based on previous research, Vizi et al. (2023) demonstrated the existence of two hepcidin-25 isoforms (hepcidin-25α and hepcidin-25β) in healthy dogs and investigated their changes in various acute and chronic inflammatory diseases [40]. Hepcidin has also been studied in multiple diseases in dogs, including portosystemic shunt, parvovirus infection, and chronic kidney disease [41–43]. The mean (± SD) serum hepcidin-25α, hepcidin-25β, and total hepcidin-25 concentrations in healthy dogs were 47.1 ± 2.4 ng/ml, 77.5 ± 3.8 ng/ml, and 124.5 ± 6.1 ng/ml, respectively [40].

Negative acute phase proteins (e.g., transferrin, cortisol-binding globulin, transthyretin, and retinol-binding protein) have serum concentrations that decrease during the acute phase reaction (APR). In all species, the most significant negative APP is albumin. During inflammation, its serum concentration may be reduced secondary to the release of IL-1, which inhibits hepatic synthesis [3, 8, 9, 44].

Acute pancreatitis (AP) is a relatively common inflammatory condition in dogs that initiates an acute-phase response, characterized by elevated serum CRP levels [45]. CRP and other APPs, including SAA, albumin, HAPT, and globulins, were measured in dogs with spontaneously occurring AP at the initial presentation and compared to healthy controls [46]. Serum CRP levels were elevated up to 20-fold in dogs, and SAA levels were elevated an average of 50-fold, with CRP concentrations positively correlated with SAA levels. HAPT was elevated 2-fold in dogs with AP and negatively correlated with CRP. The albumin levels were significantly decreased, and the levels of alpha-2 globulin were significantly increased in dogs with AP [46].

While some studies monitored acute-phase proteins serially during the hospitalization of dogs with acute pancreatitis (showing correlations with clinical severity and outcomes), dedicated studies that combine daily measurements of multiple acute-phase proteins with simultaneous histological analysis of pancreatic tissue are not well-documented in the veterinary literature. We recently performed an experimental study to assess the safety of a high-fat-containing tube-feeding diet on the recovery from pancreatitis [47]. In that study, blood samples from ten beagles were available before and after cerulein-induced pancreatitis, as well as during the 8 days of the study. In addition, because of the study’s primary aim, detailed post-mortem and histological results were available upon completion of the experiment.

Our study aimed to follow, over a week, the sequential changes in serum concentrations of six acute-phase proteins (CRP, SAA, HAPT, albumin, hepcidin-25α, hepcidin-25β) in dogs with experimentally induced acute pancreatitis to determine which APP most accurately reflects the disease course.

## Materials and methods

This study is a secondary analysis of serum samples and clinical and histological data originally collected in an experiment examining the impact of two different tube-feeding diets on the clinical progression of cerulein-induced pancreatitis [47]. Further details on the experimental setup can be found in our previously published manuscript [47]. Briefly, pancreatitis was induced by repeated cerulein injections in ten female beagle dogs aged between 1.5 and 2 years. Dogs were randomly assigned to two groups based on the fat content of their tube-feeding diet. Apart from this, their treatment, including crystalloid infusion, antiemetics, and analgesics, was identical across groups. The dogs underwent daily clinical, laboratory, and ultrasound assessments over 8 days. Blood samples were collected at baseline, then at 6, 12, and 24 h after the initial six cerulein injections, and then daily for 7 days. Daily sampling was performed after an approximately 12-hour fast (water was provided ad libitum). Cerulein administration was repeated on the 5th and 6th days (approximately 12 h apart) because clinical and laboratory signs appeared to normalize by then, raising concerns that the severity of pancreatitis might not be sufficient to draw meaningful conclusions at the study’s end. Sampling on day 5 was performed before cerulein administration, and on day 6, it was performed 12 h after day 5 administration and immediately before day 6 administration. The dogs were euthanized on the 9th day to facilitate detailed post-mortem and histological examinations of their organs to identify potential adverse effects of the higher-fat diet. Histopathology was performed by colleagues from the Department of Pathology at the University of Veterinary Medicine, Budapest. The severity of inflammation, edema, and fat necrosis in the three pancreatic regions was scored using the scoring system by De Cock et al. (2007) [48]. The histological changes in the pancreatic regions were summed to generate edema or inflammation scores. Additionally, combined edema and inflammation scores, as well as the most severe scores, were used for comparison with laboratory parameters [47].

### Diagnostics

From the collected blood samples, routine hematological and biochemistry parameters were measured, including amylase, DGGR-lipase, quantitative canine pancreas-specific lipase (cPL), total cholesterol, iron (Fe), iron-binding capacity, CRP, and albumin. Additionally, in line with this study’s aims, serum amyloid A (SAA), haptoglobin (HAPT), and hepcidin-25 alpha and beta levels were also assessed.

A reference laboratory conducted the measurements^1^: haematology was analyzed using Advia 2120 and microscopy of the stained blood smear; biochemistry was performed with a Beckman Coulter AU480 analyzer (Brae, USA), utilizing reagents for cPL by IDEXX (Kornwestheim, Germany), CRP by Randox CRP (Crumlin, Ireland) calibrated for dogs, and HAPT by Tridelta haptoglobin (Maynooth, Ireland) as previously described [29, 49–51]. SAA was measured with an immunoturbidimetric assay (LZ Vet-SAA, Eiken Chemical Co., Tokyo, Japan) on the same chemistry analyzer [28]. All study parameters, including serum CRP, SAA, HAPT, and albumin, were tested in fresh samples and measured individually on the same day as collection. Samples were kept refrigerated at 4 °C until and during transport to the laboratory. Additionally, we stored serum samples from each blood collection for hepcidin analysis, which was performed 34 months after the study concluded. Samples were kept at -80 °C until hepcidin measurements and were delivered directly from storage to the lab on dry ice. Analysis was conducted using liquid chromatography/tandem mass spectrometry (LC-MS/MS), as described in Vizi et al.’s (2023) study [40]. Unfortunately, due to a sampling error, stored samples for hepcidin measurements were unavailable from the last day of the study.

### Statistical analysis

For this research, individual data from all dogs, regardless of treatment group, were analyzed together. Descriptive statistics and line graphs were used to describe and assess overall trends in the study results. Correlations between the studied parameters were calculated by a web application using repeated measures correlation (RMCorrShiny. (n.d.). Retrieved January 8, 2025, from https://lmarusich.shinyapps.io/shiny_rmcorr) [52]. When cPL values exceeded the laboratory lower (30 ug/L) or upper (2000 ug/L) detection limits, 29 and 2001 were used for calculations. The distribution of last day’s CRP, SAA, pancreatic enzymes, and histological score values was examined using the Shapiro-Wilk test for normality. Pearson or Spearman correlation coefficient was calculated between these parameters, depending on the data distribution. Correlation coefficients below 0.3 were considered low; those between 0.3 and 0.7 were considered moderate; and coefficients above 0.7 indicated strong correlations. Data were analyzed using the Numiqo Online Statistics Calculator (numiqo e.U. Graz, Austria. URL https://numiqo.com). Because of multiple comparisons, P-values below 0.001 were considered statistically significant.

## Results

### Pancreatic enzymes

At baseline, all pancreatic enzymes were within the laboratory reference range. At 6 h after the sixth dose of cerulein injections, all dogs had amylase and lipase values above the reference range. Nine of ten dogs also had an elevated cPL above the level suggestive of pancreatitis (above 400 µg/l), and seven were above the upper limit of detection (above 2000 µg/l). The 10th dog had a borderline elevation in its cPL level (273 µg/L). However, it still represented a 7.5-fold increase compared to its own baseline value. This confirms that the induction of pancreatitis was successful. All three pancreatic enzymes started to decline after the first day’s 6th-hour peak in all but one dog, where the cPL enzyme level persisted above the upper limit of detection until the fourth day. However, even in this dog, amylase and lipase levels decreased gradually after the peak on the first day. All three pancreatic enzymes returned within the reference range by day 5, except for one dog, when the cerulein injection was repeated once on day 5 and day 6. These two bolus injections again increased all three pancreatic enzymes, but the magnitude of the change was much less pronounced. In three dogs, the lipase and cPL levels did not exceed the upper reference value. In addition, cPL levels remained below 400 µg/l in six dogs. After these second peaks on the sixth day, all three pancreatic enzymes quickly returned to normal levels by the eighth day in all dogs (Table 1). Amylase and lipase showed a very strong, significant correlation with each other: repeated measures correlation coefficient (rrm) = 0.90, 95% confidence interval (95% CI) [0.857, 0.935], *p* < 0.001). They also showed a strong correlation with the cPL levels (rrm = 0.76, 95% CI [0.663, 0.839], *p* < 0.001 for amylase, and rrm = 0.72, 95% CI [0.605, 0.807], *p* < 0.001 for lipase).

**Table 1:** Progression of amylase, lipase, cPL, CRP, SAA, haptoglobin, hepcidin-α and -β, and albumin in a canine cerulein-induced pancreatitis model. (*n* = 10)

Parameter	0 h	6 h	12 h	24 h	48 h	72 h	96 h	120 h	144 h	168 h
Amylase (100–1200 U/L)	789 ± 126	9869 ± 8400	4276 (5162)	1573 (1499)	962 (357)	811 (186)	760 (204)	1275 ± 385	746 ± 131	799 ± 125
Lipase (8–81 U/L)	21 ± 6	777 (594)	286 (171)	54 (49)	37 (21)	22 (17)	16 (12)	101 (107)	16 (13)	30 ± 17
cPL (< 200 ug/L)	47 ± 16	2001 (1001)	850 ± 570	128 (153)	76 (69)	55 (24)	48 (34)	321 (415)	39 (29)	36 (34)
CRP (< 10 mg/L)	1.7 (0.8)	5.2 (2.8)	22 ± 10	24 (25)	15 (16)	16 (12)	45 ± 38	57 ± 49	31 (45)	45 ± 37
SAA (mg/L)	0.8 ± 0.5	1.1 (0.4)	3.2 (6.5)	4.6 (12.4)	1 (7.5)	2.3 (4.5)	4.3 (40)	23 (208)	8.5 (117)	12 (151)
HAPT (g/L)	1.7 (1.4)	1.2 ± 0.2	1.2 (0.4)	1.2 (0.1)	1.2 (0.2)	1.5 (0.1)	1.4 ± 0.1	1.4 (0.2)	1.5 ± 0.1	1.5 ± 0.1
HEPC A (5.3–36.4 ng/mL)	41 ± 8.1	41 ± 8.8	41 ± 12	49 ± 12	47 ± 13	67 ± 26	54 ± 18	43 ± 5.0	4 ± 8.3	-
HEPC B (ng/mL)	91 ± 14	91 ± 16	100 (9.1)	101 ± 12	97 (9.7)	109 ± 20	101 ± 11	89 ± 8.7	87 ± 14	-
Albumin (25–41 g/L)	35 ± 1.8	35 (1.8)	36 ± 3.1	35 ± 1.8	35 ± 1.9	34 ± 1.7	33 ± 1.7	32 ± 1.5	34 ± 1.9	31 ± 2.7

The values are expressed as mean ± SD or median (IQR) depending on their distribution. The measurement units and laboratory reference values (if available) are in the left column. When cPL values exceeded the laboratory lower (30 ug/L) or upper (2000 ug/L) detection limits, 29 and 2001 were used for calculations

### Histopathology

At the end of the study, all dogs had a macroscopically intact pancreas, except for one animal with an erythematous and edematous pancreas. During histology, most dogs showed changes affecting less than 25% of their pancreatic lobes or body. Histological changes consisted only of edema in four dogs and only inflammation in two animals; both alterations were observed in three individuals, while one dog had no histological changes in the pancreas. Pancreas necrosis was not seen in any of the animals.

When comparing the last day’s pancreas enzyme levels with the histological scores, lipase and cPL did not correlate with the total inflammation scores. Meanwhile, amylase showed only a weak, nonsignificant correlation (*r* = 0.3, *p* = 0.401). In contrast, a strong but statistically nonsignificant correlation was observed between total edema scores and lipase activity (*r* = 0.7, *p* = 0.024). cPL showed a moderate, nonsignificant correlation (*r* = 0.41, *p* = 0.242), whereas amylase did not correlate with the edema scores.

### Acute phase proteins

**Fig. 1:**
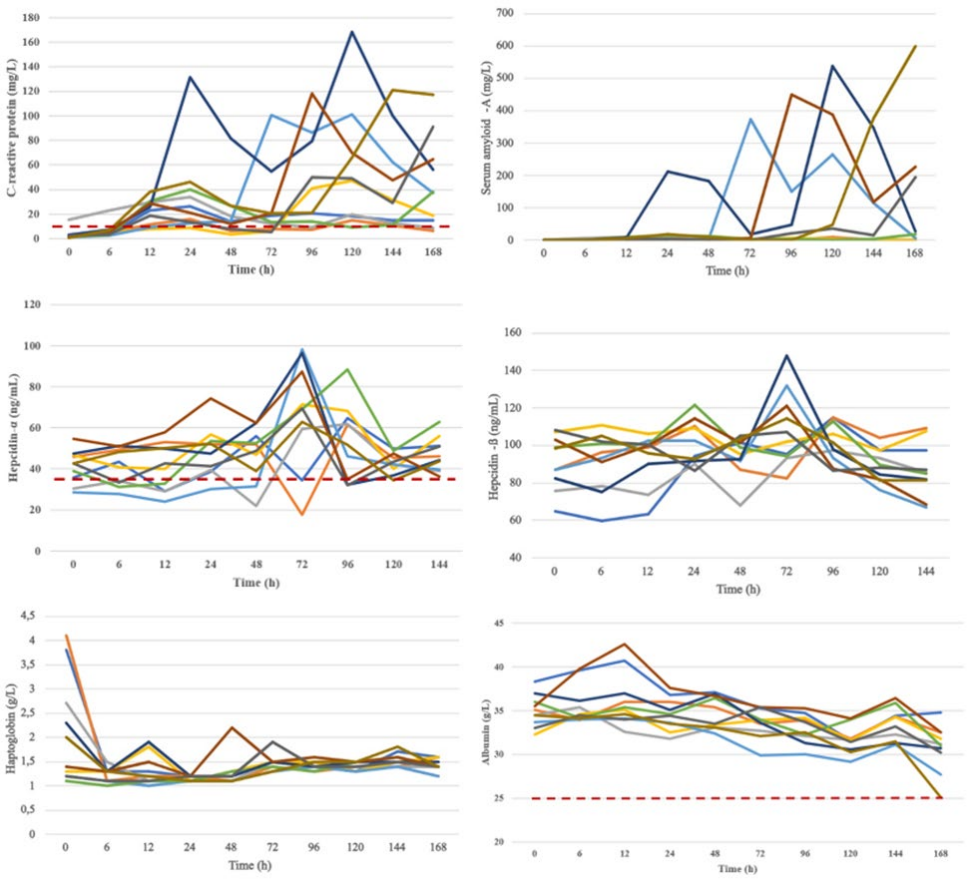
Changes in C-reactive protein (CRP), serum amyloid-A (SAA), hepcidin-α and -β, haptoglobin, and albumin levels in ten dogs during the study. Each line represents an individual dog. The red dashed line indicates the upper limit of the reference interval (RI) for CRP and hepcidin-α, and the lower limit of the RI for albumin

All measured APP values were within the reference range at baseline except for one dog with slightly elevated CRP (15.7 mg/l) and two others with mildly elevated haptoglobin (3.8 and 4.1 g/l) concentrations. The individual variations of the APP concentrations throughout the study are shown in Fig. 1, while the average values are listed in Table 1.

**Fig. 2:**
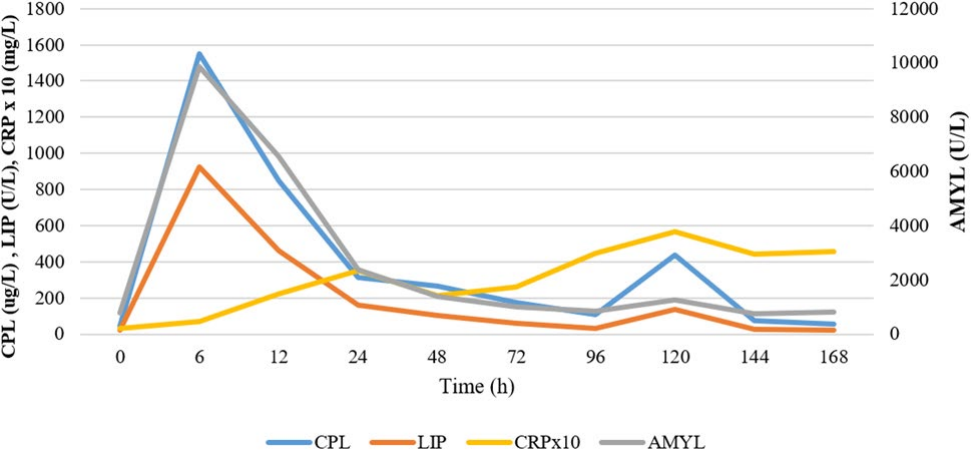
Changes in average amylase (AMYL), lipase (LIP), quantitative canine pancreas-specific lipase (CPL), and C-reactive protein (CRP) levels. The graph shows the changes in these parameters over the course of the study. Daily CRP averages have been multiplied by 10 to enhance visibility

**Fig. 3:**
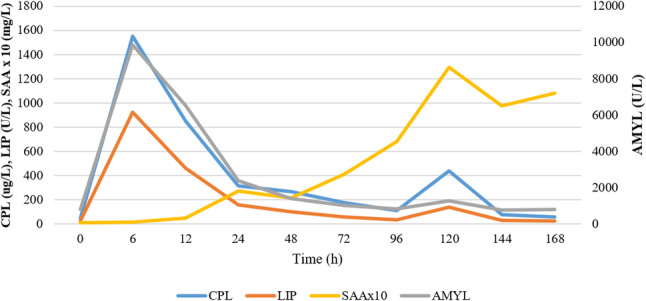
Changes in average amylase (AMYL), lipase (LIP), quantitative canine pancreas-specific lipase (CPL), and serum amyloid-A (SAA) levels. The graph shows the changes in these parameters over the course of the study. Daily averages for SAA have been multiplied by 10 to enhance visibility

CRP increased very early, usually within 6 h after administration. CRP levels showed an initial peak on the second day of the study (24 h) in 7 out of 10 dogs (Fig. 1). However, one of these seven dogs had a peak CRP value within the reference range (9.1 mg/l). The initial peak in CRP levels was observed as early as 12 h in two dogs, whereas in another dog, both CRP and SAA reached their first peak only on the fourth day (72 h). The first peak in SAA levels coincided with the first peak in CRP levels in 6 of 10 dogs. In comparison, no significant initial increase in SAA was noted in the remaining four dogs despite elevated CRP levels. CRP (and, if elevated, SAA) levels began decreasing after the initial peaks, followed by a second, larger rise after day 4 or 5 in 7 out of 10 dogs. In one dog, the second rise started only on the seventh day, while in two dogs, it had already begun by the third day. The second CRP peak coincided with the highest SAA level in 7 out of 10 dogs. In one dog, the highest SAA level peaked a day later than CRP, whereas no relevant SAA elevations were observed in two dogs. The maximum CRP change was a median 35-fold increase (range 2- to 173-fold) compared to baseline. The maximum change in SAA from the first measurement varied widely, ranging from a 2-fold to a 4494-fold increase (median 173-fold). The repeated cerulein injections on days 5 and 6 did not coincide with the tendency to initiate a second CRP and SAA elevation in most dogs (Figs. 2 and 3). No correlation was found between pancreatic enzyme levels and CRP or SAA; however, a strong positive correlation was observed between CRP and SAA (rrm = 0.85, 95% CI [0.782, 0.899], *p* < 0.001). Additionally, moderate correlations were observed between serum cholesterol levels and both CRP (rrm = 0.57, 95% CI [0.411, 0.694], *p* < 0.001) and SAA (rrm = 0.53, 95% CI [0.37, 0.667], *p* < 0.001). The CRP and SAA levels on the last day showed a statistically non-significant moderate correlation with the histological pancreatic inflammatory scores (*r* = 0.55, *p* = 0.0979, and *r* = 0.62, *p* = 0.053, respectively). However, no correlation was observed between histological edema scores and CRP or SAA levels.

The highest serum albumin levels were observed 12 h after the initial cerulein injection in 8 out of 10 dogs, and serum albumin gradually declined in all dogs throughout the study. Nevertheless, none of the dogs had serum albumin levels drop below the lower reference value. Serum albumin demonstrated a moderate negative correlation with CRP (rrm= -0.52, 95% CI [-0.657, -0.354], *p* < 0.001) and SAA (rrm= -0.51, 95% CI [-0.65, -0.343], *p* < 0.001). The Spearman correlation between the pancreas histological inflammation score and the serum CRP/albumin ratio on the last day was not significant (*r* = 0.55, *p* = 0.97). Similarly, correlations between the last day’s serum albumin or CRP/albumin ratio and other histological parameters were weak or insignificant.

**Fig. 4:**
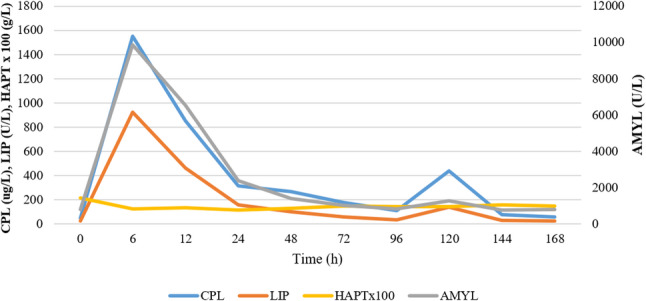
Comparison of the average amylase (AMYL), lipase (LIP), quantitative canine pancreas-specific lipase (CPL), and haptoglobin (HAPT) levels. The graph shows the changes in these parameters during the study. The daily HAPT averages have been multiplied by 100 to improve visibility

Haptoglobin for each dog decreased at different rates from the initial values after the cerulein injections, and by day 3, the values were in similar range (1.1–1.6 g/L, only showing small, irregular fluctuations. None of these fluctuations exceeded the reference value during the study. Haptoglobin did not correlate with the other measured blood parameters or histological scores (Fig. 4).

**Fig. 5:**
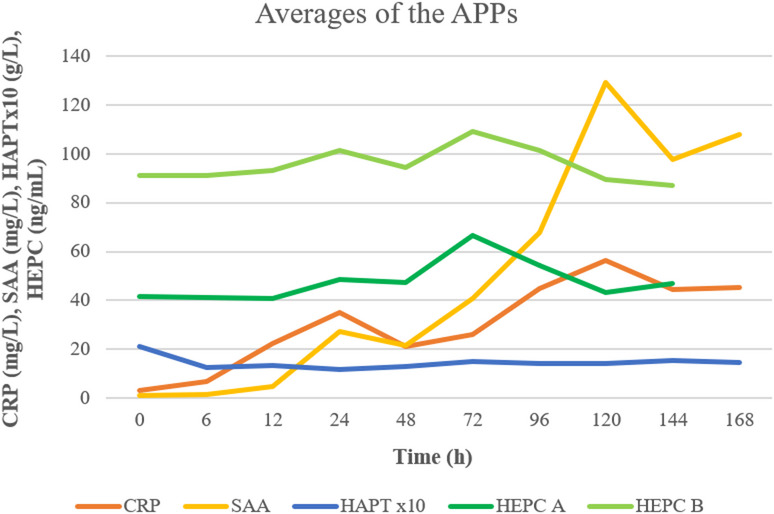
Averages of acute phase proteins in canine pancreatitis model. CRP: C-reactive protein, SAA: serum amyloid-A, HEPC A: hepcidin-α, HEPC B: hepcidin-β, HAPT: haptoglobin. Haptoglobin values have been multiplied by 10 for better visibility

The HEPC α and HEPC β values fluctuated throughout the study with some individual differences. However, they mostly changed concurrently, as shown by a moderate positive correlation (rrm = 0.62, 95% CI [0.47, 0.742], *p* < 0.001) between the two hepcidin isoforms (Fig. 5). There was no relationship between the hepcidins and the other studied APPs, pancreatic enzymes, or histological scores. There was also no link between hepcidins and iron levels or iron-binding capacities. At the beginning of the study, the hepcidin isoforms (in 1 out of 10 dogs for Hepcidin-α and in 7 out of 10 dogs for Hepcidin-β) and total hepcidin levels (in 6 out of 10 dogs) were higher than the normal range previously established by Vizi et al. (2023) in a small group of healthy dogs [40].

## Discussion

Our research was the first to investigate and compare six APPs in parallel over a week in an experimentally induced acute pancreatitis model, and also the first to examine the two forms of hepcidin in canine pancreatitis. As this was an experimental study, we had access to all dogs’ post-mortem and histological data. Thus, a direct comparison could also be made between the blood parameters and the histopathological results.

CRP is the most commonly used inflammation marker in dogs and is also used to track disease progression. Several studies investigated the use of CRP in dogs with spontaneously occurring pancreatitis. In hospitalized patients, CRP levels measured on days 3 and 4 of hospitalization differed significantly between animals that survived and those that died, suggesting that it may aid in monitoring disease progression and prognosis [53]. On the contrary, Kuzi et al. (2020) found that although CRP values were elevated at admission in hospitalized dogs with pancreatitis and that higher serum CRP concentrations were associated with higher disease severity scores, CRP was not predictive of death [54]. In another study, Keany et al. (2021) found that measured CRP values were not always statistically correlated with existing clinical impression-based scoring systems. This was likely because the patients had other pathological processes in addition to pancreatitis, and the study period did not cover the full time required for CRP to normalize, which is typically 3–14 days. However, previous studies still suggest that measuring CRP levels may be suitable for objectively monitoring changes in patient status [55]. Oberholtzer et al. (2023) found that measurements of cPLI levels > 600 µg/L and CRP > 10 mg/L in dogs with pancreatitis were associated with an increased risk of hospitalization or death [56].

CRP responded similarly to what has been observed in naturally occurring pancreatitis cases, increasing rapidly after cerulein administration and with the onset of inflammation in all subjects. This change was observed very early, as early as 6 h after administration, with a peak on the second day (24 h) in almost all cases. A decreasing trend over the following days, consistent with applied treatments, paralleling improvement in their clinical condition, was observed. Another peak in serum CRP was observed on the sixth day in some dogs after the second cerulein administration, consistent with observations after the first administration. These findings are similar to those of Holm et al. (2004), who demonstrated significantly elevated CRP levels in spontaneously developing pancreatitis compared with the control group and also showed a decrease in CRP levels parallel to improvement in the pathological condition [45]. However, in some dogs in our study, the second CRP elevation occurred before the second cerulein administration, and in others, no elevation was observed. This finding, along with the fact that CRP did not correlate with any of the measured pancreatic enzyme levels, can be explained by CRP’s nonspecific inflammatory response (as with other acute-phase proteins), meaning it can be elevated anywhere in the body, not just in the pancreas. Our study in this respect corroborates the findings of Keany et al. (2021) [55]. In our research, this phenomenon is best illustrated by the dog, which had a histologically normal pancreas but elevated CRP and SAA levels at the end of the study. Despite these considerations, CRP levels in most dogs followed the biphasic pancreatic inflammatory response in this experiment, confirming previous findings that monitoring CRP levels can track the disease course in canine pancreatitis (Fig. 2).

Previous studies showed significant increases in SAA concentration from 2 h to 5 days after an intravenous dose of E. coli lipopolysaccharide or an experimentally induced parvovirus infection [57, 58]. In the latter study, the maximum peak concentration was reached in a week [3]. In our study, the magnitude of SAA concentration elevation was variable, sometimes (but not always) exceeding that of the CRP increase, as previously demonstrated in a former study [15]. Despite a uniform animal group and the same method used to induce pancreatitis, the timing and extent of SAA elevation varied. In some dogs, it increased significantly as early as 12–24 h after induction, whereas in others it did so only after 3–4 days or later. Neither the onset of elevation nor the peak value appeared to correlate with the level of pancreatic enzyme elevation (Fig. 3). Based on our findings, SAA appears to be less suitable than CRP for monitoring the progression of acute pancreatitis in dogs. Still, its role in diagnosing systemic inflammation in spontaneous pancreatitis needs further studies.

Both CRP and SAA showed moderate correlations with total cholesterol levels in our study. The link between inflammation and cholesterol levels is a recognized but not fully understood phenomenon in human medicine. Evidence shows interdependent pathways linking cholesterol transport to chronic inflammation in conditions such as human psoriasis and systemic lupus erythematosus [59, 60]. Milanović et al. (2018) found that dogs infected with Babesia canis had increased SAA levels and decreased cholesterol and HDL fractions, along with increased HDL particle size [61]. Several pathophysiological processes and relationships are hypothesized to underlie these changes; however, none of these hypotheses have been confirmed, and further studies will be necessary in this area. Our findings support the need for continued research on CRP, SAA, and cholesterol levels in dogs with spontaneous pancreatitis.

Ceron et al. (2023) compared CRP, HAPT, and albumin levels in bitches with pyometra or pancreatitis and healthy dogs. CRP and HAPT levels increased significantly, while albumin levels decreased significantly in dogs with pancreatitis or pyometra compared to the healthy control group [62]. Kogika et al. (2003) measured serum haptoglobin, ceruloplasmin, and α-acid glycoprotein levels in healthy and leukopenic dogs with hemorrhagic gastroenteritis caused by canine parvovirus infection. They found a significant difference in all studied APPs compared to the control group [63].

The irregular fluctuations in serum haptoglobin concentrations we observed, and the absence of any spike in the values after the first or second cerulein administration, raise the question of whether these fluctuations are associated with ongoing pancreatitis (Fig. 4). Since previous studies have shown significant increases in HAPT in several diseases, we can infer that either mild pancreatitis in our study was not severe enough to cause a notable increase in its levels, or HAPT may not be a reliable marker of systemic inflammation during pancreatitis in dogs [33–35, 62, 64].

Albumin levels were within the reference range in all dogs in our study. Still, a clear declining trend was observed, and albumin was moderately and inversely correlated with CRP and SAA concentrations. The low level of systemic inflammation and the relatively short duration of our study likely explain why albumin levels remained within the reference range. Gori et al. (2020) determined CRP in dogs hospitalized with a diagnosis of acute pancreatitis and found that a higher CRP-to-albumin ratio was associated with a significant mortality risk [65]. Fabretti et al. (2020) calculated a reference range of 0.36–0.60 for the CRP/albumin ratio using a control group of 40 dogs. They also noted that this ratio was a better marker of disease severity and length of hospital stay in dogs than CRP and albumin analyzed separately [66]. The CRP-to-albumin ratio showed no better correlation with the pancreas histological scores in our study than CRP alone; however, our dogs had relatively mild pancreatitis, and albumin levels decreased only slightly during follow-up, which may explain our findings.

To our knowledge, no veterinary studies have investigated changes in serum hepcidin levels in acute pancreatitis. Olinder et al. (2022) studied serum hepcidin levels in human ICU patients to differentiate between severe sepsis/septic shock and non-septic conditions. They also evaluated the potential usefulness of hepcidin levels compared to other biomarkers for sepsis. Hepcidin levels at admission were significantly higher in the sepsis group than in the non-sepsis group. Hepcidin showed higher diagnostic accuracy for septic shock than heparin-binding protein, although it was less accurate than CRP and procalcitonin [67]. In another human study, Arabul et al. (2013) compared hepcidin and CRP levels, white blood cell (WBC) count, and multi-detector computed tomography in predicting pancreatitis severity. Hepcidin had a strong correlation with CRP and WBC in patients with acute pancreatitis and proved to be a better predictor of the severity than CRP and WBC [68].

In a previous veterinary study, levels of TNF, interleukin-6 and − 1, SAA, CRP, haptoglobin, hepcidin, and iron were measured and compared between puppies with parvoviral gastroenteritis and healthy puppies on four occasions over 14 days. Hepcidin, TNF, IL-1, IL-6, SAA, HAPT, and CRP levels were significantly higher than in the healthy control group, and a notable decrease was observed after medical treatment. They indicated that hepcidin could be used to evaluate the prognosis of sepsis [42]. Another canine study compared the two hepcidin-25 isoforms in 47 dogs with acute or chronic inflammation caused by various factors and in 14 healthy controls. It confirmed that inflammation led to a statistically significant increase in the average concentration of both serum hepcidins [40].

Neither hepcidin isoforms nor total hepcidin values correlated with clinical and laboratory alterations during our study, nor did we find any significant correlation between CRP and either hepcidin isoform. The fact that, unlike CRP and SAA, hepcidin is mainly induced by IL-6 and not by IL-1-like cytokines might explain this lack of correlation [7]. Vizi et al. (2020) also found no correlation between CRP and hepcidins in their healthy dog populations [69]. We also found no correlation between hepcidins and iron levels or iron-binding capacities, which also corroborates the findings of Vizi et al. (2020) in normal dogs. Bhamarasuta et al. (2021) compared hepcidin levels in dogs with chronic kidney disease (CKD) with those in healthy controls. They also monitored the changes in hepcidin and iron metabolism after darbepoetin treatment. Plasma hepcidin concentration did not differ between the control and CKD groups, or between the responder and nonresponder subgroups, before and after darbepoetin alfa treatment [43]. In conclusion, based on our findings, we can’t recommend using hepcidin as a marker of inflammation in dogs with acute pancreatitis.

While this study provides valuable insights into the different acute-phase responses during canine pancreatitis, it has some limitations that should be acknowledged. Pancreatitis was experimentally induced with cerulein, resulting in a transient, mild form of the disease that may not fully mirror the acute-phase responses observed in more severe natural cases. Although we combined data from two groups of dogs from a previous study and analyzed them as a single population (10 dogs), the sample size remained small. Additionally, the animals were fed two different diets for the purposes of our previous study, which could have influenced the responses of some acute-phase proteins.

## Conclusions

During our experimental study, CRP was the most consistent marker for tracking temporal changes in pancreatic enzymes among the studied APPs. Further research is recommended to investigate the clinical use of SAA as a biomarker in canine pancreatitis. The relationships between cholesterol metabolism, CRP, and SAA levels also warrant additional investigation. Our findings did not support the practical usefulness of hepcidins or haptoglobin for monitoring pancreatitis in dogs. Because our study was exploratory, further research on naturally occurring cases is needed to better understand the prognostic significance of various APPs in canine pancreatitis.

## Data Availability

The datasets used and/or analysed during the current study are available from the corresponding author on reasonable request.

## Abbreviations

AGP: α1–acid glycoprotein
AP: Acute pancreatitis
APP: Acute phase protein
APR: Acute phase response
CI: Confidence interval
CRP: C–reactive protein
HAPT: Haptoglobin
HEPC: Hepcidin
HDL: High–density lipoprotein
Hgb: Haemoglobin
IL-1, IL-4, IL-6, IL-8, IL-11, IL-13: Interleukin-1, interleukin-4, interleukin-6, interleukin-8, interleukin-11, interleukin-13
LAT: Latex agglutination turbidimetric immunoassay
rrm: Repeated measures correlation coefficient
RBC: Red blood cell count
RI: Reference interval
SAA: Serum amyloid–A
SRMA: Steroid–responsive meningitis–arteritis
TNFα: Tumor necrosis factor alpha
WBC: White blood cell count

## Footnotes

1 Praxislab Kft., Iskola u. 3., 1038 Budapest, Hungary.
